# Adenosine A_2A_ receptor mediates hypnotic effects of ethanol in mice

**DOI:** 10.1038/s41598-017-12689-6

**Published:** 2017-10-04

**Authors:** Teng Fang, Hui Dong, Xin-Hong Xu, Xiang-Shan Yuan, Ze-Ka Chen, Jiang-Fan Chen, Wei-Min Qu, Zhi-Li Huang

**Affiliations:** 1 Department of Pharmacology and Shanghai Key Laboratory of Bioactive Small Molecules, School of Basic Medical Sciences, State Key Laboratory of Medical Neurobiology, Institutes of Brain Science and Collaborative Innovation Center for Brain Science, Fudan University, Shanghai, 200032 China; 2 Department of Neurology, School of Medicine, Boston University, Boston, Massachusetts USA

## Abstract

Ethanol has extensive effects on sleep and daytime alertness, causing premature disability and death. Adenosine, as a potent sleep-promoting substance, is involved in many cellular and behavioral responses to ethanol. However, the mechanisms of hypnotic effects of ethanol remain unclear. In this study, we investigated the role of adenosine in ethanol-induced sleep using C57BL/6Slac mice, adenosine A_2A_ receptor (A_2A_R) knockout mice, and their wild-type littermates. The results showed that intraperitoneal injection of ethanol (3.0 g/kg) at 21:00 decreased the latency to non-rapid eye movement (NREM) sleep and increased the duration of NREM sleep for 5 h. Ethanol dose-dependently increased NREM sleep, which was consistent with decreases in wakefulness in C57BL/6Slac mice compared with their own control. Caffeine (5, 10, or 15 mg/kg), a nonspecific adenosine receptor antagonist, dose-dependently and at high doses completely blocked ethanol-induced NREM sleep when administered 30 min prior to (but not after) ethanol injection. Moreover, ethanol-induced NREM sleep was completely abolished in A_2A_R knockout mice compared with wild-type mice. These findings strongly indicate that A_2A_R is a key receptor for the hypnotic effects of ethanol, and pretreatment of caffeine might be a strategy to counter the hypnotic effects of ethanol.

## Introduction

Ethanol is one of the most highly abused psychoactive compounds worldwide^1,2^. It produces a variety of acute and chronic effects^3^, which have a significant socio-economic impact on the individuals, their families, and society. Ethanol can cause premature disability and death, accounting for an estimated 6–9% of all deaths^4,5^. Extensive clinical studies have documented that acute ethanol has a profound impact on sleep^4,6^, and acute discontinuation of alcohol in alcoholics results in severe disturbance of sleep architecture^6–8^. In addition, these sleep impairments are so severe that they are primary predictors of relapse in recovering alcoholics^9,10^. Thus, it is of paramount importance to identify the mechanism underlying the effects of ethanol on sleep-wake regulation. However, the central mechanisms involved in sleep-wake regulation by ethanol remain elusive.

Adenosine, a potent sleep-promoting substance^11^, is a key mediator of many behavioral and neuronal responses to ethanol^12–15^. Dysregulation of adenosine signaling has been implicated in ethanol-use disorders^14–18^. Ethanol is known to increase adenosine release^19,20^ and decrease adenosine uptake by inhibiting the type 1 equilibrative nucleoside transporter, which result in increased extracellular adenosine^21–23^. Accumulated extracellular adenosine induces sleep by activating adenosine A_1_ receptor (A_1_R) and A_2A_ receptor (A_2A_R) in the central nervous system^24,25^. Among adenosine receptors responsible for sleep induction, the role of A_2A_R is predominant in sleep regulation. Administration of the selective A_2A_R agonist CGS21680 to the subarachnoid space adjacent to the basal forebrain and lateral preoptic area reliably induces a dramatic increase in non-rapid eye movement (non-REM, NREM) sleep, whereas infusion of A_1_R agonists produces weak and variable effects^26–29^. Furthermore, homeostasis of sleep-wake regulation is unaltered in animals lacking A_1_R^30^. However, the role of A_2A_R in ethanol-induced hypnotic effects is still in debate.

Caffeine, another widely used psychoactive compound^31^, binds A_1_R and A_2A_R with similar affinity as a receptor antagonist. The antagonistic role of caffeine in the ethanol-induced hypnotic effects is controversial. Some studies have suggested that caffeine offsets ethanol-induced changes in information processing, memory, and psychomotor performance^32–35^. However, other studies have been unable to confirm these results^36,37^. Therefore, it is unclear whether caffeine can block ethanol-induced hypnotic effects.

In the present study, we characterized sleep-wake profiles of C57BL/6Slac mice after an intraperitoneal (i.p.) injection of ethanol and found that ethanol increased NREM sleep in a dose-dependent manner. Furthermore, we demonstrated an antagonistic role of caffeine in hypnotic effects of ethanol. Pretreatment but not post-treatment of caffeine abolished the hypnotic effects of ethanol. Because caffeine reduces sleep by blocking A_2A_R^38,39^, we assessed possible involvement of A_2A_R in the hypnotic effects of ethanol using A_2A_R knockout (KO) and wild-type (WT) mice. After ethanol administration, A_2A_R WT mice showed an increase in duration of NREM sleep, but A_2A_R KO mice showed no change in time spent in NREM sleep. These findings indicate that A_2A_R plays an important role in the hypnotic effects of ethanol. Understanding the molecular mechanism underlying the hypnotic effects of ethanol may provide new therapeutic approaches for treating alcoholism and blocking acute behavioral impairment due to ethanol.

## Results

### Ethanol increased NREM sleep and decreased wakefulness in C57BL/6Slac mice

**Figure 1:**
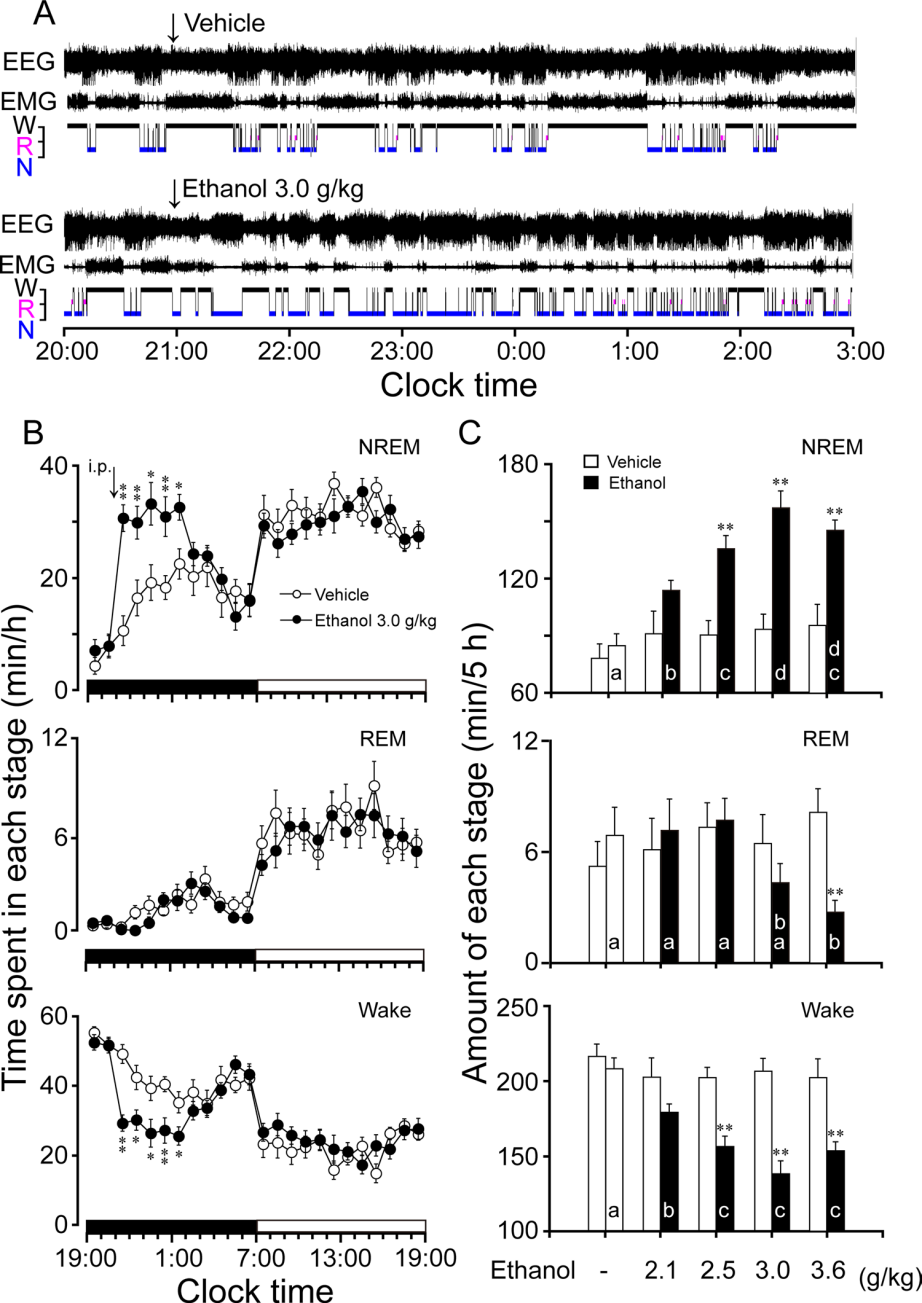
Sleep-wake profiles produced by administration of ethanol in C57BL/6Slac mice. (**A**) Typical examples of polygraphic recordings and corresponding hypnograms illustrating changes of sleep over 7 h (20:00–03:00) following vehicle (upper panel) or ethanol (lower panel) administration. (**B**) Changes in NREM sleep, REM sleep, and wakefulness in mice treated with 3.0 g/kg ethanol. (**C**) Dose-response effects on total time spent in NREM sleep, REM sleep, and wakefulness for 5 h after administration of vehicle and ethanol. Values are mean ± SEM (n = 9–10). (**B**) *P < 0.05 or **P < 0.01 indicates significant differences compared with their own control as assessed by two-way ANOVA followed by Fisher’s least-significant difference (PLSD) test. (**C**) **P < 0.01 indicates significant differences compared with the control as assessed by non-paired, two-tailed Student’s *t* test. The letters “a, b, c, d” indicate different subsets among doses of ethanol as assessed by one-way ANOVA followed by Duncan’s multiple range test.

To investigate the hypnotic effects of ethanol, electroencephalograms (EEG) and electromyograms (EMG) were recorded for 2 consecutive days in C57BL/6Slac mice. On day 1, the mice were treated with vehicle i.p. at 21:00 in the early phase of the dark (active) period, and the recordings made on that day served as the own control. The animals were then treated with vehicle or ethanol (2.1, 2.5, 3.0, 3.6 g/kg, i.p.) 24 h later. Because mice spend most of their time sleeping during the light period, it is more difficult to evaluate effects of a drug on duration of sleep in the light period than in the dark period. Therefore, the experiments were performed during the dark period when animals spend most of their time in wakefulness. Typical examples of polygraphic recordings and corresponding hypnograms from one mouse given vehicle or 3.0 g/kg ethanol are shown in Fig. 1A. The mouse treated with ethanol spent more time in NREM sleep compared with their own control.

Time course changes revealed that ethanol at 3.0 g/kg significantly increased NREM sleep (F_1,198_ = 21.3, *P* < 0.05) and decreased wakefulness (F_1,198_ = 18.0, *P* < 0.05) in C57BL/6Slac mice compared with their own control (Fig. 1B). Ethanol at 3.0 g/kg increased NREM sleep for 5 h following ethanol administration, which was consistent with a reduction in wakefulness during the same period. The effects began within the first hour after ethanol injection and last for 5h. There was no further disruption of sleep architecture during the subsequent period. No time course changes on REM sleep were observed.

Total time spent in NREM sleep, REM sleep, and wakefulness were measured for 5 h after ethanol injection, because 3 g/kg ethanol increased NREM sleep for 5 h. Ethanol dose-dependently increased NREM sleep (F_4,47_ = 17.9, *P* < 0.01) and reduced wakefulness (F_4,47_ = 15.6, *P* < 0.01) (Fig. 1C). Ethanol at 2.5, 3.0, and 3.6 g/kg increased the total duration of NREM sleep by 1.5-, 1.8-, and 1.6-fold (*P* < 0.01), respectively, which was consistent with a reduction in wakefulness by 23%, 33%, and 24% (*P* < 0.01), respectively, compared with their own control in each group. However, ethanol at 2.1 g/kg did not affect the cumulative amount of NREM sleep when measured for 5 h after injection. In contrast, ethanol at 3.6 g/kg reduced REM sleep by 67% when measured for 5 h after ethanol injection (Fig. 1C). However, the other doses of ethanol did not affect the duration of REM sleep. These results clearly indicate that ethanol increases NREM sleep in a dose-dependent manner and reduces REM sleep at a high dose of 3.6 g/kg.

### Ethanol shortened sleep latency and altered sleep architecture in C57BL/6Slac mice

**Figure 2:**
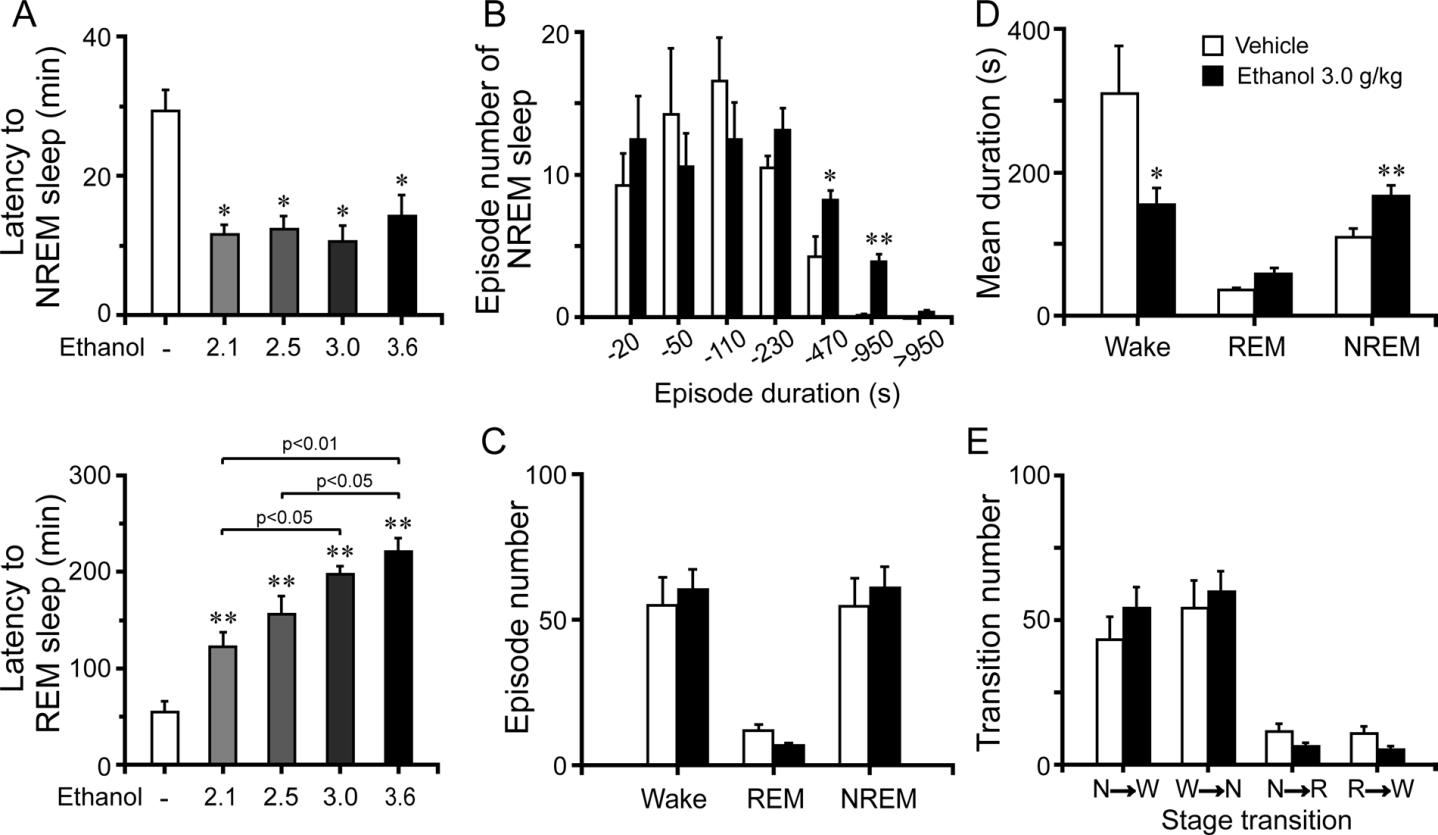
Changes in sleep latency and architecture produced by administration of ethanol. Effect of ethanol on NREM and REM sleep latency (**A**). Numbers of NREM sleep bouts (**B**), total episode numbers (**C**), mean durations (**D**), and stage transitions (**E**) during the first 5 h after administration 3.0 g/kg ethanol. Values are mean ± SEM (n = 10). (**A**) *P < 0.05 or **P < 0.01 indicates significant differences assessed by one-way ANOVA followed by Fisher’s PLSD test. (**B**–**E**) *P < 0.05 or **P < 0.01 indicates significant differences performed using non-paired, two-tailed Student’s *t* test.

To assess initiation of the sleep state after injection, we measured the latencies to NREM and REM sleep, which were defined as the time from vehicle or ethanol injection to the first appearance of a NREM or REM sleep episode that lasted for at least 20 s^40^. As shown in Fig. 2A (upper panel), injection of ethanol remarkably shortened NREM sleep latency. The latencies to NREM sleep in mice treated with ethanol (2.1, 2.5, 3.0, and 3.6 g/kg) were 11.6, 12.3, 10.6, and 14.2 min, respectively, which were markedly shorter than the latency of 29.4 min after vehicle injection (*P* < 0.05). The short NREM sleep latency following ethanol injection clearly indicates that ethanol accelerates initiation of NREM sleep. In addition, REM sleep latency was dose-dependently prolonged by ethanol (Fig. 2A, lower panel). The latencies to REM sleep in mice treated with ethanol (2.1, 2.5, 3.0, and 3.6 g/kg) were 122.4, 155.8, 197.2, and 221.4 min, respectively, which were longer than the latency of 55 min after vehicle injection (Fig. 2A, lower panel). Prolonged REM sleep latency in ethanol-injected mice clearly indicates that ethanol inhibits initiation of REM sleep.

To better understand the changes in sleep architecture caused by 3 g/kg ethanol, we determined the episode number and mean duration of NREM sleep, REM sleep, and wakefulness, as well as stage transitions between the 3 vigilance stages. Ethanol at 3.0 g/kg increased the number of bouts of NREM sleep with durations of 230–950 s (Fig. 2B). There was no difference in the number of episodes of wakefulness, NREM sleep, and REM sleep (Fig. 2C). In addition, the mean duration of NREM sleep increased by 53% (Fig. 2D, *P* < 0.01), with a concomitant 50% decrease in wakefulness (Fig. 2D, *P* < 0.05). The mean transition numbers showed no change during the 5 h immediately following administration of ethanol (Fig. 2E). These results suggest that ethanol increases bouts of longer NREM sleep and mean duration of NREM sleep, which extend the overall duration of NREM sleep.

### Pretreatment with caffeine offset ethanol-induced hypnotic effects

**Figure 3:**
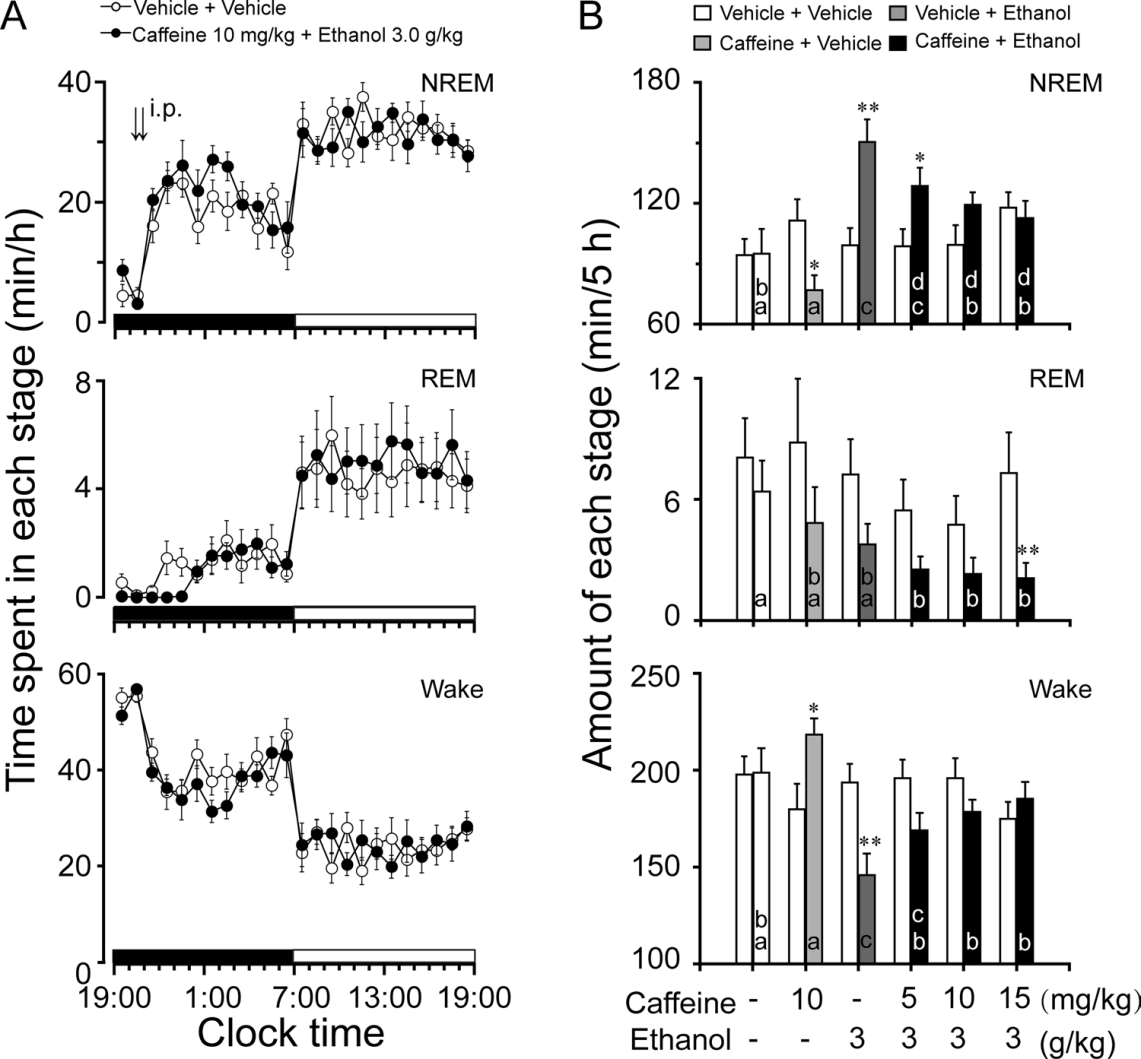
Sleep-wake profiles produced by ethanol administration with caffeine pretreatment in C57BL/6Slac mice. (**A**) Changes in NREM sleep, REM sleep, and wakefulness in mice treated with 10 mg/kg caffeine and 3.0 g/kg ethanol. (**B**) Dose-response effect on total time spent in NREM sleep, REM sleep, and wakefulness for 5 h after administration of vehicle and drugs. Values represent mean ± SEM (n = 10). (**A**) Comparisons of time course changes in the hourly amounts of each stages assessed by two-way ANOVA followed by Fisher’s PLSD test. (**B**) *P < 0.05 or **P < 0.01 indicates significant differences compared with their own control as assessed by two-tailed unpaired Student’s *t* test. The letters “a, b, c, d” indicate different subsets among doses of ethanol as assessed by one-way ANOVA followed by Duncan’s multiple range test.

It is well known that caffeine induces wakefulness by blocking A_2A_R^39^. To determine whether caffeine reduces hypnotic effects of ethanol, caffeine (5, 10, or 15 mg/kg, i.p.) was administered to C57BL/6Slac mice 30 min prior to ethanol injection. As shown in Fig. 3A, pretreatment with caffeine at a dose of 10 mg/kg completely abolished the hypnotic effects induced by ethanol 3 g/kg when compared with their own control (F_1,198_ = 2.3, *P* = 0.15).

Total time spent in NREM sleep, REM sleep, and wakefulness was calculated for 5 h after administration of caffeine and ethanol. Ethanol with vehicle pretreatment increased the duration of NREM sleep by 1.5-fold, which was consistent with a 25% decrease in wakefulness (Fig. 3B, *P* < 0.01). Caffeine alone increased the duration of wakefulness by 1.2-fold, which was consistent with a 32% decrease in NREM sleep (Fig. 3B, *P* < 0.05). Pretreatment with caffeine at 10 mg/kg or 15 mg/kg 30 min before ethanol did not increase the cumulative amount of NREM sleep when measured for 5 h after injection, compared with their own control. When caffeine was pretreated at 5 mg/kg, ethanol still increased the total duration of NREM sleep (Fig. 3B, *P* < 0.05). These findings indicate that pretreatment with caffeine dose-dependently reduces hypnotic effects of ethanol.

**Figure 4:**
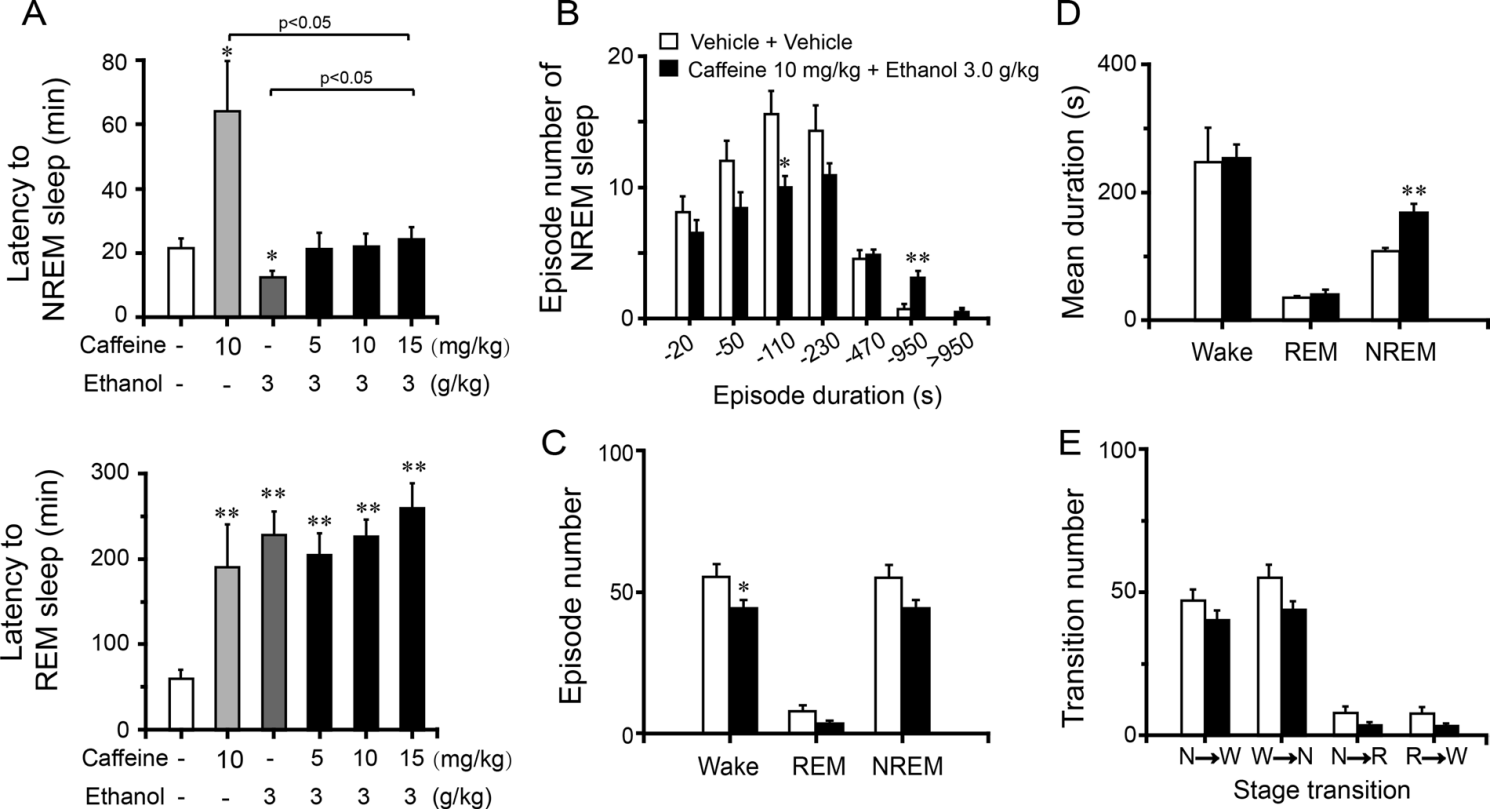
Changes in sleep latency and architecture produced by administration of ethanol with pre-treatment of caffeine. Effect of ethanol on NREM and REM sleep latency (**A**). Numbers of NREM sleep bouts (**B**), total episode numbers (**C**), mean durations (**D**), and stage transitions (**E**) during the first 5 h after administration of 10 mg/kg caffeine and 3.0 g/kg ethanol. Values are mean ± SEM (n = 10). (**A**)*P < 0.05 or **P < 0.01 indicates significant differences assessed by one-way ANOVA followed by Fisher’s PLSD test. (**B**–**E**) *P < 0.05 or **P < 0.01 indicates significant differences performed using two-tailed unpaired Student’s *t* test.

The latencies to NREM and REM sleep were also determined. As shown in Fig. 4A (upper panel), injection of caffeine with vehicle prolonged the latency to NREM sleep and injection of ethanol with vehicle pretreatment remarkably shortened NREM sleep latency. The latency to NREM sleep in mice treated with vehicle and ethanol was 12.5 min, which was markedly shorter than the latency of 24.8 min in vehicle control (*P* < 0.05). However, there were no significant differences in NREM latency in response to vehicle administration with vehicle pretreatment versus ethanol administration with caffeine pretreatment at 5, 10, and 15 mg/kg. Pretreatment with caffeine at 15 mg/kg 30 min before injection of ethanol abolished the decrease in NREM sleep latency induced by ethanol with vehicle pretreatment (Fig. 4A, upper panel). These results indicate that caffeine pretreatment counteracts initiation of NREM sleep induced by ethanol.

Following pretreatment with vehicle or caffeine (5, 10, and 15 mg/kg), the latencies to REM sleep in mice treated with ethanol were 233.1, 204.7, 225.8, and 259.7 min, respectively, which were longer than the latency of 60.1 min after vehicle administration with vehicle pretreatment. And caffeine 10 mg/kg with vehicle also prolonged the latency to REM sleep compared with vehicle control (Fig. 4A, lower panel). These results clearly indicate that caffeine pretreatment does not alter the increase in REM sleep latency induced by ethanol.

Analysis of sleep architecture showed that pretreatment with 10 mg/kg caffeine 30 min before administration of 3.0 g/kg ethanol decreased the number of bouts of NREM sleep with durations of 60–110 s (*P* < 0.05) and increased the number of bouts of NREM sleep with durations of 480–950 s (Fig. 4B, *P* < 0.01). The number of episodes of NREM sleep was reduced by 20% (Fig. 4C, *P* = 0.054), and the mean duration of NREM sleep increased by 55% (Fig. 4D, *P* < 0.01). These factors resulted in no difference in the total amount of NREM sleep. Furthermore, there were no differences in the mean duration of REM sleep and wakefulness (Fig. 4D). In contrast, ethanol with caffeine pretreatment reduced the episode number of wakefulness (Fig. 4C, *P* < 0.05), and the episode number of REM sleep did not change (Fig. 4C). ﻿The stage transition numbers showed no significant change following administration of ethanol with caffeine pretreatment (Fig. 4E)﻿.﻿These results show that pretreatment with 10 mg/kg caffeine completely abolishes the hypnotic effects caused by 3.0 g/kg ethanol. However, caffeine does not completely block ethanol-induced impairment of sleep architecture.

### Ethanol still increased NREM sleep and decreased wakefulness with post-treatment of caffeine

**Figure 5:**
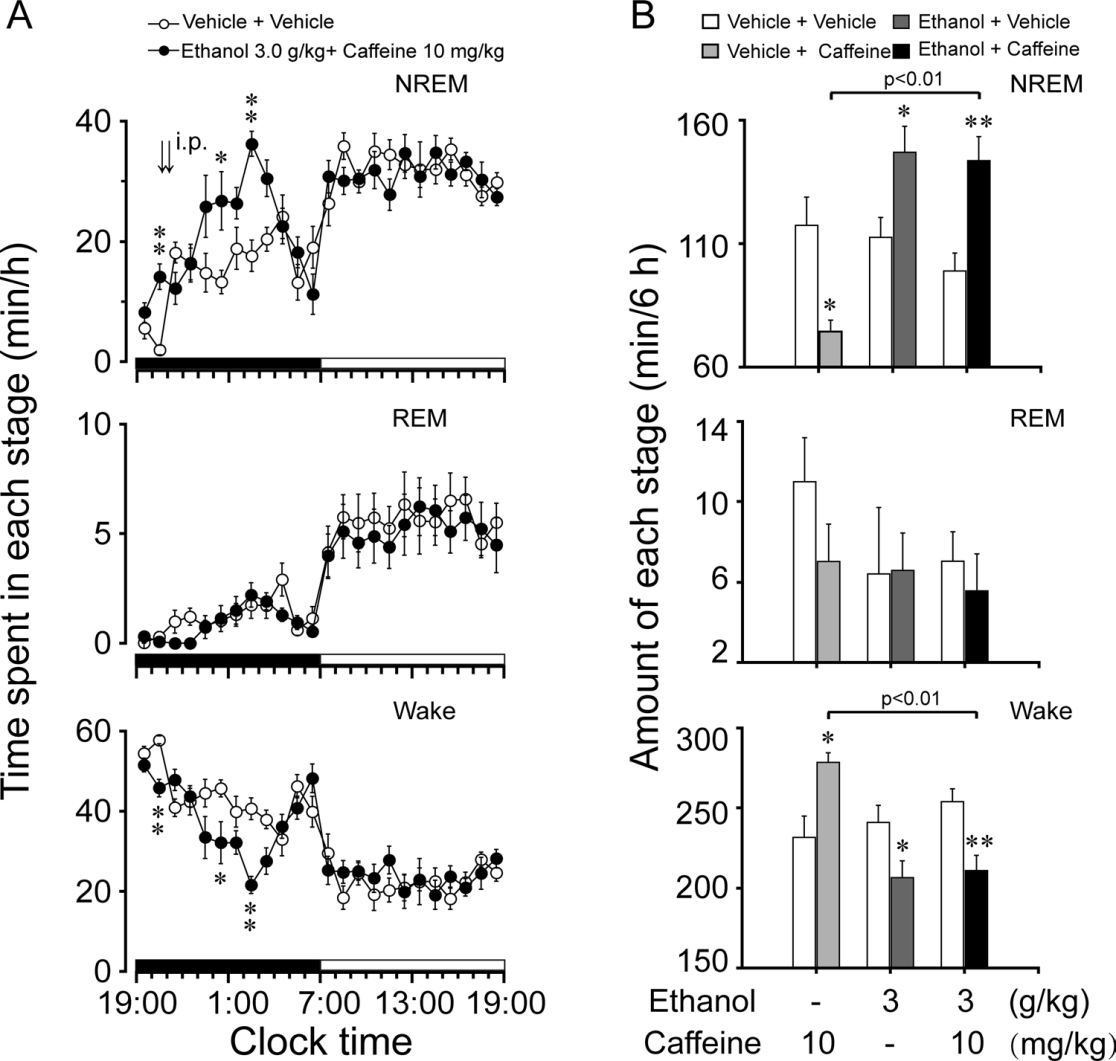
Sleep-wake profiles produced by administration of ethanol with post-treatment of caffeine in C57BL/6Slac mice. (**A**) Changes in NREM sleep, REM sleep, and wakefulness in mice treated with 3.0 g/kg ethanol and 10 mg/kg caffeine. (**B**) Total time spent in NREM sleep, REM sleep, and wakefulness for 6 h after administration of vehicle and drugs. Values are mean ± SEM (n = 4–8). *P < 0.05 or **P < 0.01 indicates significant differences compared with their own control as assessed by two-way ANOVA followed by Fisher’s PLSD test (**A**), or by two-tailed unpaired Student’s *t* test with their own control and one-way ANOVA among groups (**B**).

To determine whether post-treatment with caffeine alters the hypnotic effects of ethanol, we administered ethanol (3.0 g/kg) at 20:30, followed by caffeine (10 mg/kg) administration at 21:00 into C57BL/6Slac mice. Ethanol with post-treatment of caffeine still increased NREM sleep (F_1,158 _= 12.6, *P* < 0.05) and decreased wakefulness (F_1,158_ = 10.5, *P* < 0.05) compared with their own control (Fig. 5A). Ethanol at 3.0 g/kg increased NREM sleep at 20:30–21:00 before administration of caffeine (*P* < 0.01, Fig. 5A). With post-treatment of caffeine at 10 mg/kg, ethanol at 3.0 g/kg still significantly increased NREM sleep by 2.0- and 2.1-fold (*P* < 0.05) during the fourth and sixth hours after injection, respectively, compared with control, with decreases in wakefulness by 30% and 47% (*P* < 0.05), respectively (Fig. 5A). There was no further disruption of sleep architecture during the subsequent period. In addition, we calculated the total time spent in NREM sleep, REM sleep, and wakefulness for the 6-h period following administration. Vehicle with post-treatment of caffeine decreased the total amount of NREM sleep by 37%, with a 20% increase in wakefulness (Fig. 5B). However, ethanol with or without post-treatment of caffeine increased the total amount of NREM sleep by 1.5-fold and 1.3-fold, with a 17% and 14% decrease in wakefulness, respectively (Fig. 5B). There were no statistical differences between the two groups. These results indicate that post-treatment of caffeine can not offset ethanol-induced hypnotic effects.

### Deletion of A_2A_R abolished the hypnotic effects of ethanol

**Figure 6:**
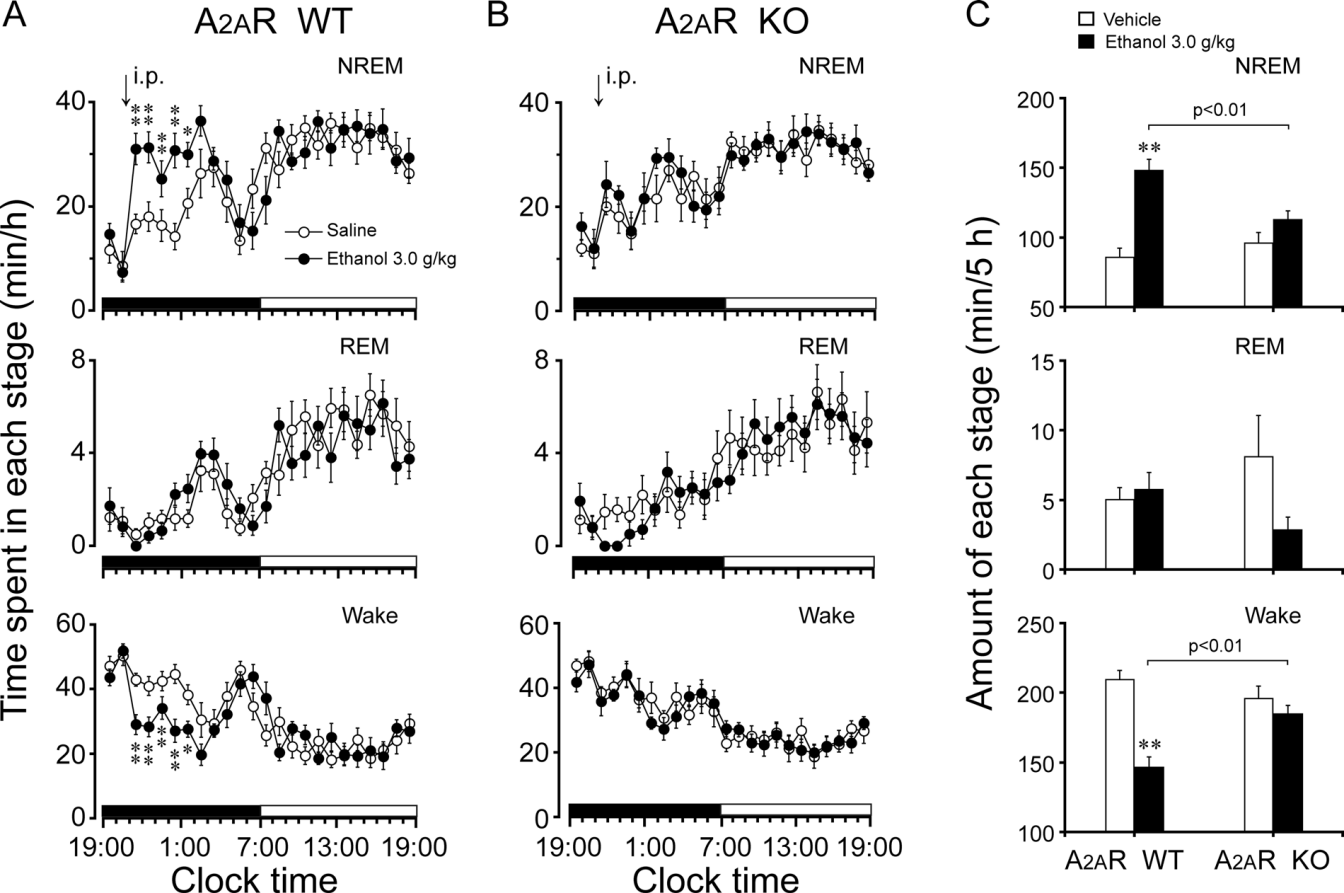
Sleep-wake profiles produced by administration of ethanol in A_2A_R WT and A_2A_R KO mice. (**A,B**) Changes in NREM sleep, REM sleep, and wakefulness in A_2A_R WT (**A**) and KO (**B**) mice treated with 3.0 g/kg ethanol. (**C**) Effect of ethanol on total time spent in NREM sleep, REM sleep, and wakefulness for 5 h after administration of vehicle and ethanol in A_2A_R WT and KO mice. Values are mean ± SEM (n = 8–9). (**A,B**) *P < 0.05 or **P < 0.01 indicates significant differences compared with their own control as assessed by two-way ANOVA followed by Fisher’s PLSD test. (**C**) **P < 0.01 indicates significant differences compared with their own control as assessed by two-tailed unpaired Student’s *t* test. *P* < 0.01 indicates significant differences between A_2A_R WT and KO mice performed using two-tailed unpaired Student’s *t* test.

To clarify the importance of A_2A_R in the hypnotic effects of ethanol, we used A_2A_R KO mice and their WT littermates. Ethanol given to A_2A_R WT mice at 3 g/kg significantly increased NREM sleep for 5 h (F_1,106_ = 30.7, *P* < 0.01) compared with their own control (Fig. 6A), which was consistent with a reduction in wakefulness (F_1,106_ = 30, *P* < 0.01). However, A_2A_R KO mice given 3 g/kg ethanol did not exhibit any significant change in duration of NREM sleep compared with their own control (Fig. 6B). In A_2A_R WT mice, for the first 5 h after the ethanol injection, the total duration of NREM sleep increased by 1.7-fold, which was consistent with a 30% decrease in wakefulness, compared with their own control. However, there were no differences in the duration of NREM sleep and wakefulness in the A_2A_R KO mice (Fig. 6C). Compared with the A_2A_R KO mice, ethanol increased NREM sleep by 1.3-fold and decreased wakefulness by 21% in A_2A_R WT mice (Fig. 6C). These results clearly indicate that A_2A_R is a key receptor involved in ethanol-induced hypnotic effects.

### Ethanol altered the power spectra of NREM sleep in mice

**Figure 7:**
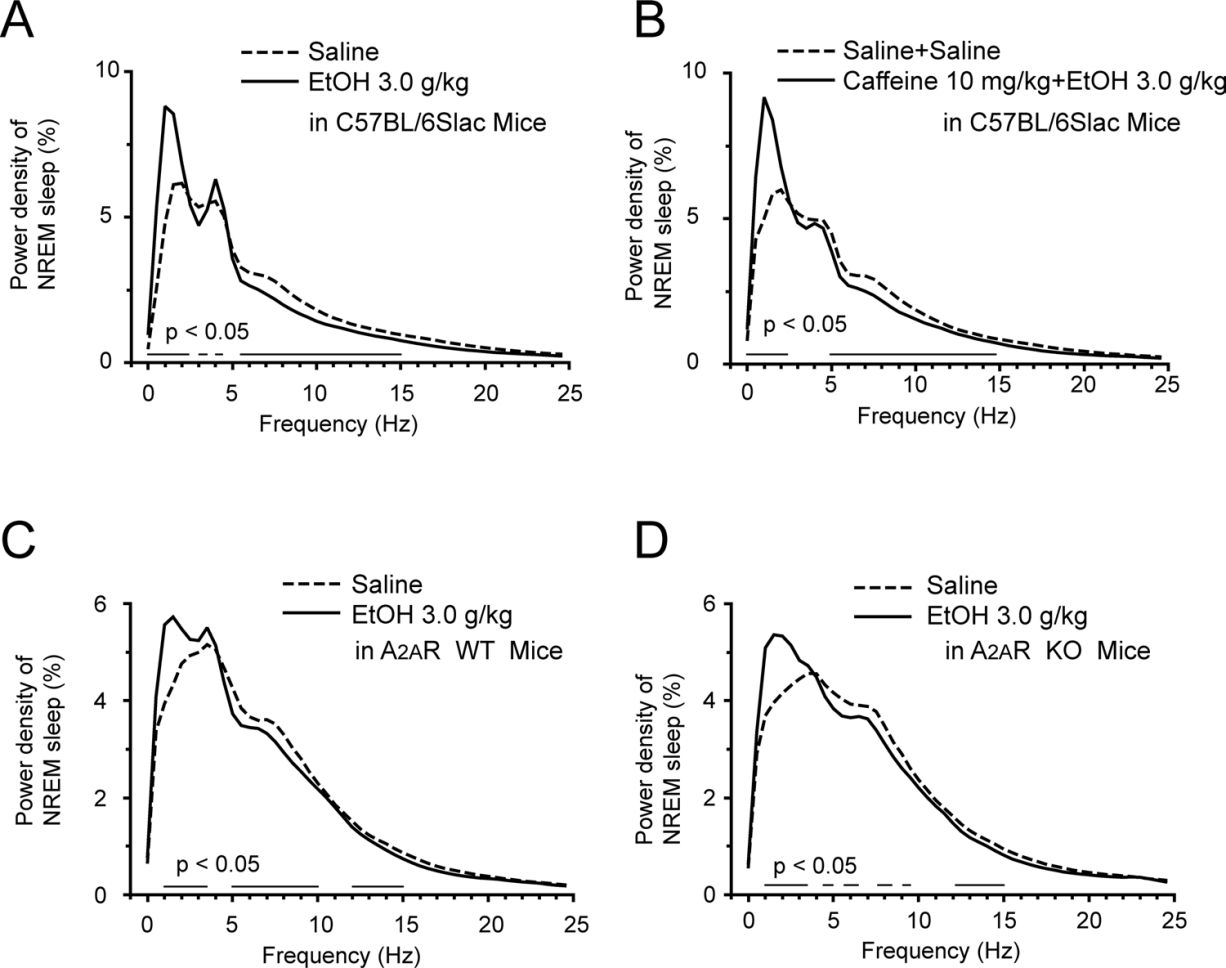
Characteristics of EEG power density of NREM sleep after administration of vehicle or treatment. (**A**) EEG power density curves during NREM sleep after administration of vehicle or ethanol in C57BL/6Slac mice. (**B**) EEG power density curves during NREM sleep after administration of vehicle or caffeine and ethanol in C57BL/6Slac mice. (**C,D**) EEG power density curves during NREM sleep after administration of vehicle or ethanol in A_2A_R WT (**C**)﻿ and A_2A_R KO ﻿(**D**)﻿ mice. Horizontal bars indicate location of a statistically significant difference. Values are mean ± SEM (n = 8–10). *P < 0.05 indicates significant differences compared with their own control as assessed by two-tailed unpaired Student’s *t* test.

The delta activity (0.5–4 Hz) during NREM sleep is a reliable indicator of sleep need^41,42^. We evaluated the EEG power spectra and compared the power densities of vehicle and treatment in mice during NREM sleep in each experiments. The power of each 0.5 Hz bin was first averaged across the sleep stages individually and then normalized by calculating the relative duration of each bin from the total power (0–24.5 Hz) for each individual animal. As shown in Fig. 7A, compared with their own control, ethanol (3.0 g/kg) elevated delta power density in the frequency ranges of 0.5–2, 2.5–3 and 3.5–4 Hz during NREM sleep. With caffeine pretreatment, ethanol (3.0 g/kg) still elevated delta power density in the frequency ranges of 0.5–2 Hz during NREM sleep (Fig. 7B). And ethanol elevated delta power density in A_2A_R WT and A_2A_R KO mice during NREM sleep (Fig. 7C, D). These results indicated that ethanol increased delta power density, which could not completely offset by caffeine or genetic knockout of A_2A_R.

## Discussion

Ethanol consumption is an integral part of daily life in many societies, as it can produce positive mood states and has stress-relieving effects^43^. Furthermore, ethanol is one of the most commonly used “over the counter” sleep aids, although consuming large amounts of alcohol clearly has the potential for enormous detrimental impacts, including sleep disruption. However, the molecular mechanisms that underlie the hypnotic effects of ethanol remain poorly identified. In the present study, we found that ethanol dose-dependently increased NREM sleep by increasing the mean duration of NREM sleep and shortening the latency to NREM sleep, which is consistent with other studies^4,6,44^. In addition, ethanol dose-dependently prolonged the latency to REM sleep and decreased the amount of REM sleep. Taken together, these results indicate that ethanol affects sleep-wake behaviors by altering sleep architecture.

Acute intoxicating effects of ethanol may be related to GABA facilitation and glutamate inhibition, which are also critically involved in sleep-wake regulation^6^. However, the pharmacological effects of ethanol involve multiple mechanisms, so other targets may be relevant to the effects of ethanol. In the present study, 3.0 g/kg ethanol significantly increased NREM sleep for 5 h in WT mice. However, these hypnotic effects were completely abolished in adenosine A_2A_R KO mice, indicating that adenosine A_2A_R is essential for the hypnotic effects of ethanol.

In the central nervous system, adenosine is an important endogenous purine neuromodulator that modulates many important cellular processes in neurons. Adenosine is proposed to act as a homeostatic regulator of sleep in which levels in the brain rise during waking and decline during NREM sleep^24^. Extracellular levels of adenosine depend on rates of formation, degradation, and diffusion between intracellular and extracellular spaces^45^. It has been reported that ethanol inhibits the type 1 equilibrative nucleoside transporter, which reduces adenosine reuptake and thereby increases extracellular adenosine^22^, and adenosine synthesis is increased during ethanol metabolism^46^. Furthermore, ethanol may act directly in the brain to increase extracellular adenosine^20^. Ethanol-increased extracellular levels of adenosine may contribute to its hypnotic effects. We found that caffeine, an adenosine receptor antagonist, can abolish the hypnotic effects of ethanol. Taken together, these results suggest that adenosine is a key mediator in the hypnotic effects of ethanol.

The delta activity (0.5–4 Hz) during NREM sleep is a reliable indicator of sleep depth. Compared with their own control, ethanol increased the delta power in the mice, which means the NREM sleep induced by ethanol was not physiological sleep. Pharmacological antagonism or genetic deletion of A_2A_R did not reverse the increased delta power density induced by ethanol. It has been reported that GABA facilitation and glutamate inhibition induced by ethanol are critically involved in sleep-wake regulation^6^. However, the mechanism of delta power change induced by ethanol remains to be elucidated.

There are 4 adenosine receptor subtypes, all of which are G protein-coupled receptors. A_1_R and A_3_R are primarily coupled to the Gi family of G proteins, whereas A_2A_R and A_2B_R are mostly coupled to the Gs type of G proteins^47^. A_2B_R is expressed widely but generally at very low levels, whereas A_3_R is expressed at intermediate levels in the hippocampus and cerebellum^48^. Little is known about the functional significance of A_2B_R and A_3_R in sleep^24^. Accumulated evidence has indicated that A_2A_R rather than A_1_R plays a key role in the effects of adenosine on sleep^24–27^. Several studies suggest that A_2A_R stimulation may be involved in the reinforcing effects of ethanol^49,50^. Caffeine is a non-specific adenosine A_1_R and A_2A_R antagonist. We found that pretreatment with 10 mg/kg caffeine completely blocked the hypnotic effects of ethanol, which was consistent with results observed in A_2A_R KO mice. These data further indicate that A_2A_R plays an important role in the hypnotic effects of ethanol.

A_2A_Rs are abundantly expressed in the striatum^51^. In our previous study, global genetic knockout of A_2A_R, but not A_1_R, abolished arousal effect of caffeine^39^, and local deletion of A_2A_R in ventral striatum blocked caffeine-induced wakefulness^38^, indicating ventral striatum A_2A_R mediate caffeine-induced wakefulness. Optogenetic or chemogentic activation of A_2A_R expressing medium spiny neurons in the ventral striatum robustly induced NREM sleep^52^. Alcohol induces adenosine release and decreases adenosine uptake, resulting in an increase extracellular level of adenosine. Taken together, we thought that caffeine can block alcohol-induced adenosine combining with striatum A_2A_ receptors.

Adenosine, as a non-classical neurotransmitter, mediates several other behavioral effects of ethanol including ataxia^16^. Ataxia appeared to be mediated by an abnormal balance between excitatory and inhibitory neurotransmitters induced by ethanol in the brain. Adenosine has been shown to modulate both GABAergic and glutamatergic transmission, and therefore adenosine maybe involved in the disruption of excitatory-inhibitory balance induced by ethanol. Pharmacological studies have shown that adenosine mediates ethanol-induced ataxia primarily through A_1_R in the whole brain^53–55^, striatum^56^, cerebellum^57^, and motor cortex^58^. But A_2A_R located mainly in the striatum maybe also involved in mediating ethanol-induced ataxia with A_2A_R agonist and antagonist altering the ethanol-induced motor incoordination^53^. However, the role of adenosine receptors in ataxia and other ethanol-induced molecular and behavioral effects need more research.

There is a popular belief that coffee can offset the debilitating effects of alcoholic intoxication^59,60^. However, several other studies could not demonstrate that the antagonistic effects of caffeine reduced performance deficits induced by ethanol^59,61^. In our experiment, pretreatment with caffeine at 10 mg/kg 30 min before injection of ethanol offset the hypnotic effects of ethanol. However, the antagonistic effects of caffeine were reduced when injected 30 min after administration of ethanol. With post-treatment of caffeine at 10 mg/kg, ethanol at 3.0 g/kg still increased NREM sleep for 2 h after administration, which was consistent with a reduction in wakefulness for 2 h. The different antagonistic effects following pretreatment or post-treatment with caffeine may result from alterations in sleep propensity. When caffeine was administered post-treatment, ethanol induced high sleep propensity in the mice. During the first 2 h, the high concentration of caffeine could offset the hypnotic effects of ethanol. However, as caffeine was metabolized, the antagonistic effects were reduced, which resulted in a resurgence of ethanol-induced hypnotic effects. In contrast, pretreatment with caffeine induced hyperarousal before ethanol injection. During low sleep propensity, the impact of caffeine metabolism may be weakened, so caffeine might completely offset the hypnotic effects of ethanol. These results indicate that the antagonistic effects of caffeine depend on whether caffeine is administered before or after ethanol intake.

The antagonistic effects of caffeine observed in this study are consistent with previous human experimental studies showing that caffeinated drinks reduce subjective feelings of ethanol intoxication, which is known as the “masking effect”^36,62^. Consumption of ethanol mixed with drinks may lead to subjective drunkenness induced by hypnotic effects, which can be delayed by caffeine during the early period. This results in an increase in ethanol consumption. Therefore, the intoxicating effects of ethanol mixed with caffeinated drinks are expected to be more severe, with a 3-fold greater risk of being legally intoxicated, in an accident, and injured during the subsequent period^34,62^. In this study, pretreatment with caffeine could reduce the hypnotic effects of ethanol, which suggests that combining ethanol and caffeinated drinks may be harmful. This is in agreement with an announcement by the U.S. Food and Drug Administration stating that caffeine is an unsafe food additive when combined with alcohol. Thus, the present results indicate that ingestion of caffeine-containing drinks with ethanol consumption is a high-risk behavior. Furthermore, A_2A_R is the most important target of caffeine^63^. A_2A_R gene KO reduced hypnotic effects of ethanol, which indicates that A_2A_R plays an important role in the mechanisms underlying the “masking effect”.

Our results indicate that ethanol dose-dependently promotes NREM sleep in mice, and A_2A_R mediates hypnotic effects of ethanol.

## Methods

### Animals

Male SPF inbred C57BL/6Slac mice (weighing 20–26 g, 11–13 weeks old) were obtained from Shanghai Laboratory Animal Center (SLAC), Chinese Academy of Sciences (Shanghai, China). C57BL/6Slac mouse is a substrain of C57BL/6J, which is got from the Jackson laboratory and inbred in SLAC laboratory. A_2A_R KO and WT littermates of the inbred C57BL/6 strain were generated from heterozygotes (provided by Boston University School of Medicine, Boston, MA). The animals were housed individually at a constant temperature (22 ± 0.5°C), with a relative humidity of 60 ± 2%, on an automatically controlled 12 h light/dark cycle (lights on at 07:00, illumination intensity ≈100 lux)^64^, and with free access to food and water. All efforts were made to minimize animal suffering and use only the number of animals required for production of reliable scientific data. All experiments were carried out in accordance with the National Institutes of Health Guide for the Care and Use of Laboratory Animals and approved by the Fudan University Committee on Animal Care and Use.

### Polygraphic recording and vigilance state analysis

Under pentobarbital anesthesia (50 mg/kg, i.p.), mice were chronically implanted with EEG and EMG electrodes for polysomnographic recordings as described previously^65–68^. One implant, which served as EEG electrodes, consisted of 2 stainless steel screws (1 mm diameter) inserted through the skull into the cortex (anteroposterior, +1.0 mm and left-right, −1.5 mm from bregma or lambda) according to the atlas of Franklin and Paxinos^69^. The other implant, which served as EMG electrodes, consisted of 2 insulated stainless steel, Teflon-coated wires bilaterally placed into both trapezius muscles. All electrodes were attached to a microconnector and fixed to the skull with dental cement.

After EEG/EMG implantation, mice were housed individually in transparent barrels for 7 d to eliminate the pain and stress from surgery. Then, mice were connected to cables and given 4 d to adapt in an insulated soundproof recording chamber before EEG/EMG recording. The animals then entered the pharmacological phase of the study. Sleep-wakefulness states were monitored for a period of 48 h, which comprised baseline and experimental days. Cortical EEG and EMG signals were amplified, filtered (EEG, 0.5–30 Hz; EMG, 20–200 Hz), digitized at a sampling rate of 128 Hz, and recorded using SLEEPSIGN (Kissei Comtec, Nagano, Japan) as described previously^39,70^. When completed, polygraphic recordings were automatically scored off-line in 10 s epochs as wakefulness, REM sleep, and NREM sleep by SLEEPSIGN according to standard criteria^39,71^. As a final step, defined sleep-wake stages were examined visually and corrected if necessary.

### Pharmacological treatments

Ethanol (20% v/v, Sigma-Aldrich, Saint Louis, MO) was administered i.p. at 21:00 on the day of the experiment at doses of 2.1, 2.5, 3.0, or 3.6 g/kg. For baseline data, mice were injected i.p. with vehicle. To test the receptor mechanism, mice were pretreated with caffeine (Alfa Aesar, Ward Hill, MA) i.p. at 5, 10 or 15 mg/kg before ethanol injection. In a separate group, mice were post-treated with 10 mg/kg caffeine 30 min after ethanol administration. The drugs were freshly prepared prior to use, and injection volume (10 mL/kg) was kept constant.

### Statistical analysis

Comparisons of time course changes in the hourly amounts of each stages in C57BL/6Slac mice treated with ethanol or vehicle, and the hourly amounts of each stages in C57BL/6Slac mice treated with combination of caffeine and ethanol or vehicle, and the hourly amounts of each stages in A_2A_R WT/KO mice treated with ethanol or vehicle were assessed using two-way ANOVA followed by Fisher’s least-significant difference (PLSD) test. Histograms of the amounts of sleep and wakefulness were assessed by one-way ANOVA followed by Duncan’s multiple range test. Significance of NREM and REM latency was assessed by one-way ANOVA followed by PLSD test. Comparisons of sleep amounts, as well as number of sleep/wake events, duration and transition of sleep/wake events between vehicle and treatment were performed using a non-paired, two-tailed Student’s *t* test. The EEG power spectra were compared using two-way ANOVA followed by PLSD.
